# Efficient transposition of Tn*4556* by alterations in inverted repeats using a delivery vector carrying a counter-selectable marker for* Streptomyces*

**DOI:** 10.1007/s10295-018-2101-x

**Published:** 2018-11-20

**Authors:** Masahiro Sota, Akiko Sakoda, Haruo Ikeda

**Affiliations:** 1 NAGASE R&D CENTER, NAGASE & CO., LTD., Kobe, Hyogo 651-2241 Japan; 2 Kitasato Institute for Life Sciences, Kitasato University, Sagamihara, Kanagawa 252-0373 Japan

**Keywords:** Class-II transposon, Counter selection, *Streptomyces*, Phenylalanyl-t-RNA synthetase

## Abstract

A 6625-base pair transposon, Tn*4556*, was initially isolated from a* Streptomyces* strain and a sequence analysis was performed; however, its annotation data remain incomplete. At least three positions were identified as frameshift and base-exchange errors by resequencing. The revised sequence revealed that Tn*4556* contains four open reading frames that encode transposase, methyltransferase, isoprenyl diphosphate transferase, and resolvase, respectively. Thirty-eight-base pair inverted repeat (IR) sequences at both ends contained a 1-bp mismatch flanked by a target duplication site, and transposition efficiency was improved by the replacement of imperfectly matched IR-L to perfectly matched IR-L. The detection of Tn*4556* transposition was markedly facilitated using a delivery vector carrying a strictly counter-selectable marker for* Streptomyces* strains.

## Introduction

Transposable elements and insertion sequences (ISs) that are capable of moving from a replicon to others are important tools in the study of bacterial genetics and gene expression. The distribution of these mobile elements in the genome is also of interest in the evolution of organisms. Some transposable elements and ISs in* Streptomyces* strains have been isolated [2, 4, 14, 19], and the distribution of ISs in the* Streptomyces* genome was recently reported [10]. Among transposons, the class-II transposable element Tn*4556* was identified in neomycin-producing *Streptomyces fradiae* in 1987 [5, 6]. Tn*4556* derivatives carrying antibiotic-resistance markers were found to be useful for transposition in* Streptomyces* strains [16, 22]. The most preferable derivative Tn*4560*, which contains the resistance marker viomycin phosphotransferase, was used to target genes involved in secondary metabolite biosynthesis [9]. The 6.8-kb sequence of Tn*4556* was elucidated in 1990 [18] and at least nine ORFs have been annotated from sequence data; however, some overlapped in both strands. Thus, since the original sequence may contain errors, we resequenced Tn*4556* to clarify actual ORFs and examined the efficient transposition of Tn*4556*.

## Results and discussion

### Resequencing of Tn*4556*

**Fig. 1:**
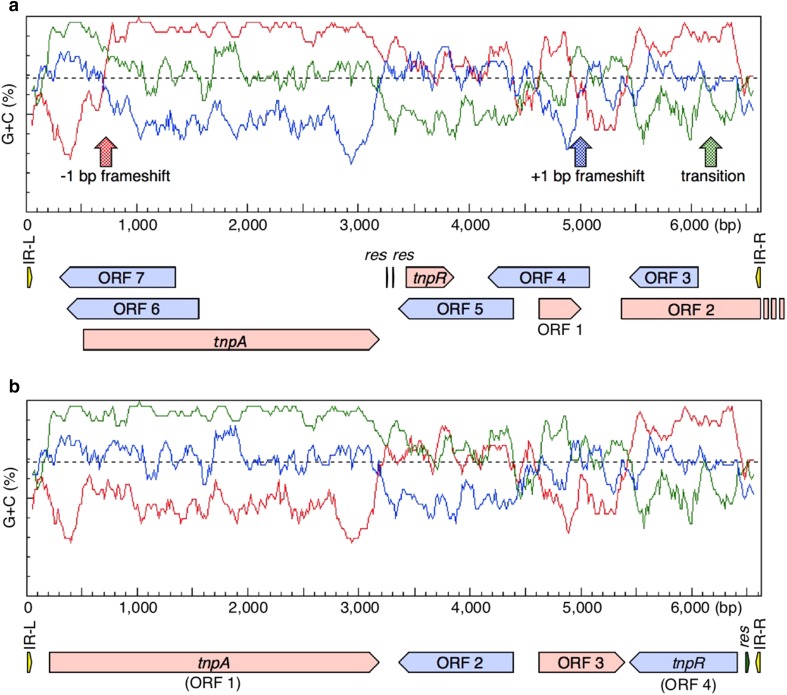
Organization of ORFs and the frame plot of Tn*4556* derived from the 6625-bp **a** original sequence and **b** revised sequence. The direction of transcription and relative sizes of the ORFs deduced from an analysis of the nucleotide sequences are indicated. A frame plot was calculated by a window size of 40 codons and step size of 5 codons. Dashed lines indicate average G + C % (68.4%). IR-L, IR-R, and *res* indicate the left end of the inverted repeat, the right end of the inverted repeat, and the internal resolution site, respectively. The genes *tnpA* and *tnpR* encode transposase and resolvase, respectively. ORF2 and ORF3 in the revised sequence **b** encode predicted methyltransferase and isoprenyl diphosphate transferase, respectively. Vertical arrows in **a** indicate different points between the original and revised sequences of Tn*4556*

A 6.64-kbp *Bam*HI fragment of Tn*4556* in pUC1232 [5], which contains the entire Tn*4556* sequence, was subcloned into pUC19 and the sequence was analyzed using a next-generation sequencer (accession # LC417441). Three positions differed from the previous sequence (accession # M29297 [15]). The first position was missing one base (a −1-bp frameshift) between 719 and 720 nt from the left end of the inverted repeat (IR-L) of the original sequence (Fig. 1a). The original ORF1 (892 aa) was annotated as a transposase (TnpA), but without the N terminus region found in other TnpAs. Based on the revised sequence (Fig. 1b), the start codon of the revised ORF1 was located upstream of the original ORF1 (from 505 to 3183 nt), and the revised ORF1 was located from 200 (TTG start) to 3184 nt (TGA stop), which encodes 994-aa TnpA (a characteristic motif of PF01526: Tn*3* transposase DDE domain was observed), and the deduced polypeptide matched other TnpAs in a BLAST analysis. Furthermore, the revised ORF2 was annotated from 4392 (TGA stop) to 3355 nt (ATG start; encoded in the complementary strand), which encodes a methyltransferase domain-containing protein (345 aa; WP_085572232 of* Streptomyces* sp. 13-12-16 shows 95% identity and 97% similarity, WP/086708490 of *S. castelarensis* shows 95% identity and 97% similarity, and WP_116427250 of *S. spongiicola* shows 95% identity and 97% similarity), in which characteristic motifs of PF13847 (methyltransferase domain: 127–236 aa), PF08241 (methyltransferase domain: 133–228 aa), and PF13649 (methyltransferase domain: 131–224 aa) were observed, and was identical to ORF5 of the original annotation (Fig. 1a, b). The second position was the insertion of one base (a + 1-bp frameshift) at 5001 nt of the original sequence (Fig. 1a) and this insertion terminated translation because the insertion of an adenine residue made the stop codon TGA. The revised ORF3 was located from 4625 (ATG start) to 5401 nt (TGA stop), which encodes putative isoprenyl diphosphate transferase (258 aa; WP_085572231 of* Streptomyces* sp. 13-12-16 shows 96% identity and 98% similarity, WP_037940392 of *S. toyocaensis* shows 96% identity and 98% similarity, and WP_116427251 of *S. spongiicola* shows 96% identity and 97% similarity), in which a characteristic motif of PF01255 (putative undecaprenyl diphosphate synthase: 31–257 aa) was observed. The final position was at 6194 nt as a guanine residue (Fig. 1a; the original sequence was adenine). This region was defined as a truncated ORF in the original annotation [18], whereas a gene was located from 5445 (TGA stop) to 6419 nt (ATG start; in the complementary strand) in this resequencing. The revised ORF4 (324 aa) was annotated as a resolvase (TnpR). A previous study reported that the downstream ORF (3420–3851 nt of the original sequence) of TnpA was defined as a TnpR (Fig. 1a; ref 16), whereas the ORF did not contain the conserved motif (PF00589; phage integrase family) found in the TnpRs of transposons; however, the revised ORF4 was defined as a TnpR because the motif for PF00589 was found at 587–971 aa by a Pfam search. Thus, the revised sequence revealed that Tn*455*6 contains four ORFs. Class-II transposons form a cointegrate intermediate with the target replicon upon transposition and the intermediate is resolved by TnpR at the internal resolution site (*res* site; in many cases, a palindrome-like structure is found in the *res* site). In the original sequence, possible *res* sites were defined between 3243 and 3251 nt, and between 3307 and 3299 nt; however, these two sequences were not found in the palindrome-like structure. On the other hand, since the region between 6497 and 6527 nt in the revised sequence formed a palindrome structure containing an 11-bp stem with a 1-bp mismatch and 9-base loop structure, this region flanking the right end of the inverted repeat (IR-R) may function as a *res* site for resolving the cointegrate intermediate during the transposition process.

### Construction of a new delivery vector carrying a counter-selectable marker for transposition

The transposition of Tn*4556* and its derivatives was efficiently performed using a delivery vector replicated in* Streptomyces* strains rather than non-replicative vectors, such as the *Escherichia coli* plasmid pUC19. After transposition, the delivery vector containing the Tn*4556* derivative has to be cured from the strain. We used the temperature-sensitive replication vector pKU110, derived from the pIJ101 replicon, for transposition in previous studies [9]; however, the elimination of the vector from the strain was not fully achievable. In the present study, we constructed a new delivery vector carrying a suicide gene for the transposition of Tn*4556* derivatives. Although some suicide genes were used for counter selection in* Streptomyces* strains, their use was limited because sensitivity depends on the strain [7, 8]. The *E. coli* phenylalanyl-t-RNA synthetase β-subunit (PheS) was useful as a counter-selectable marker because its A294G variant misincorporates 4-chlorophenylalanine into cellular proteins during translation, thereby causing cell death [17]. This counter-selectable system was also applied to other bacteria [1, 3, 11, 13, 21]. While wild-type* Streptomyces* is insensitive to 4-chloro-dl-phenylalanine (> 20 mM), we demonstrated for the first time that* Streptomyces* strains carrying an extra copy of the gene encoding the PheS variant are sensitive to 4-chloro-dl-phenylalanine. *S. avermitilis* or *S. lividans* harboring a plasmid containing mutant *pheS* (A339G; corresponding to the A294G variant of *E. coli* PheS) genes (designed from the amino acid sequence of *S. viridochromogenes* DSM 40736; accession # WP_003989071) was sensitive to ~ 2.5 mM or 10 mM 4-chloro-dl-phenylalanine, respectively, (Table 1). Since the aa position 251 (Thr) of *E. coli* PheS was involved in substrate recognition, this position was our target for the enhancement of sensitivity to 4-chlorophenylalanine [15]. After the introduction of an aa replacement in the second target at Thr278 of* Streptomyces* PheS (corresponding to Thr251 of *E. coli* PheS), sensitivity to 4-chloro-dl-phenylalanine was enhanced by approximately eightfold by the variant at T278S or T278A in PheS^A339G^ (Table 1). We then constructed the new delivery vector pGM160∆*aac1::oriT::pheS*^*A339G/T278A*^::Tn*4556*-*aac3(IV)*, and the transposition of Tn*4556*-*aac(3)IV* was efficiently performed because it was easy to isolate progeny carrying the transposon without the delivery vector after a high-temperature incubation at 37 °C and the subsequent selection of 4-chlorophenylalanine resistance.

**Table 1:** Susceptibility to 4-chloro-dl-phenylalanine of* Streptomyces* strains and their exoconjugants carrying the mutant type of the *pheS* gene

*S. avermitilis* MA-4680		4-chloro-dl-phenylalanine (µg/ml)							
PheS	0	0.156	0.313	0.625	1.25	2.5	5	10	20
Wild type	++	++	++	++	++	++	++	++	++
A339G	++	++	++	++	++	±	–	–	–
A339G/T278S	++	+	±	–	–	–	–	–	–
A339G/T278A	++	+	±	–	–	–	–	–	–

*S. lividans* TK24		4-chloro-dl-phenylalanine (µg/ml)							
PheS	0	0.156	0.313	0.625	1.25	2.5	5	10	20
Wild type	++	++	++	++	++	++	++	++	++
A339G	++	++	++	++	++	++	+	–	–
A339G/T278S	++	++	++	+	±	–	–	–	–
A339G/T278A	++	++	++	+	±	–	–	–	–

The PheS state of the wild type indicates strains harboring pGM160∆*aac1::oriT*. A339G, A339G/T278S, and A339G/T278A indicate strains harboring pGM160∆*aac1::oriT::pheS*^*A339G*^, pGM160∆*aac1::oriT::pheS*^*A339G/T278S*^, and pGM160∆*aac1::oriT::pheS*^*A339G/T278A*^, respectively. These plasmids were introduced from *E. coli* GM2929 (*dam dcm*) *hsdS::*Tn*10*/pUB307 [12] by conjugation. Exoconjugants were selected from a mixed culture by insensitive to carmonam (100 µg/ml; counterselect the sensitive *E. coli* donor) and resistance to thiostrepton (20 µg/ml). Susceptibility to 4-chloro-dl-phenylalanine was examined on YS medium (4 g of yeast extract, 10 g of soluble starch, and 20 g of agar per liter of deionized water and pH was adjusted to 7.0) containing 15 µg/ml of thiostrepton and the various concentrations of 4-chloro-dl-phenylalanine indicated. Synthetic *pheS* genes encoding PheS^A339G^, PheS^A339F/T278A^, and PheS^A339G/T278S^ (accession # LC417438, LC417439, and LC417440, respectively) were prepared by Eurofins Genomics Co., Ltd., Tokyo, Japan. Each synthetic DNA was digested with *Mlu*I/*Xba*I and the 1.46-kb fragment digested was ligated with the largest *Mlu*I/*Xba*I-pGM160∆*aac1::oriT* [12] fragment. pGM160∆*aac1::oriT::pheS* was selected

Abbreviations indicate the following: ++ preferable growth, *+* growth,  ±  slight growth (a few colonies occurred), – no growth

### Improvements in transposition using perfectly matched IR-L

**Fig. 2:**
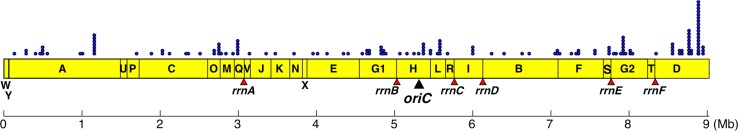
*Ase*I-map of *S. avermitilis* chromosome and transposition loci of Tn*4556*-*aac(3)IV.* The dot indicates transposition locus. The 5-bp target duplication, e.g., GGGTT, TAGAG, TGCTC, GACTG, ACCAT, GGATC, GGAGC, ATGAC, AGGTA and so on, was also confirmed at the insertion site by the sequence. Abbreviations *oriC* and *rrnA*-*F* indicate the replication origin and ribosomal RNA (16S–23S–5S rRNA) operons

In bacterial transposons, IRs at both ends of class-II transposons were relatively long (38–110 bp). Tn*4556* possessed 38-bp IRs with a 1-bp mismatch (Table 2). The exchange to the perfectly matched IRs of the transposon Tn*Had2* in the IncP-1β plasmid pUO1 from *Delftia acidovorans* strain B improved transposition frequency [20]. After the cytosine residue of the 5′-end of IR-L of Tn*4556* was replaced to a guanine residue, the modified IR-L perfectly matched to IR-R (Table 2). Tn*4556*-*aac(3)IV* carrying perfectly matched IR-L was joined to pGM160∆*aac1::oriT::pheS*^*A339G/T278A*^ and transposition efficiency was examined. As shown in Table 2, transposition efficiency was approximately five- to tenfold better than that of wild-type Tn*4556*-*aac(3)IV* and the transposition occurred randomly on *S. avermitilis* chromosome (Fig. 2).

**Table 2:** Transposition of wild-type Tn*4556*-*aac(3)IV* and its derivative consisting of perfectly matched IR-L in *S. avermitilis*

IR-L/IR-R	Transposition efficiency^a^
	5.4 × 10^−4^
	3.5 × 10^−4^
	4.2 × 10^−4^
	1.8 × 10^−3^
	3.1 × 10^−3^
	1.9 × 10^−3^

pGM160∆*aac1::oriT::pheS*^*A339G/T278A*^::Tn*4556*-*aac(3)IV* and its derivative containing perfectly matched IR-L in *E. coli* GM2929 *hsdS::*Tn*10*/pUB307 were conjugated with spores of *S. avermitilis* SUKA24 [9] on M4 medium [12] supplemented with 20 mM MgCl_2_. After incubation at 30 °C for 18 h, 1 ml of sterile water containing 2 mg/ml of carmonam and 400 µg/ml of thiostrepton was overlaid onto one plate and each plate was incubated at 30 °C for 5 days. Exoconjugants were replicated onto YMS medium [12] containing 15 µg/ml of thiostrepton and 12.5 µg/ml of nalidixic acid. After sporulation, spores on the surface of the agar plate were harvested, and spore numbers were then counted and suspended in 20 v/v % sterile glycerol (defined as thio^r^; stored at − 30 °C). Approximately 10^3^ ~ 10^4^ spores were spread onto one agar plate (antibiotic-free YMS medium) and incubated at 37 °C until sporulation. Each plate was replicated onto YS medium (see Table 1) containing 10 µg/ml of apramycin and 2.5 mM 4-chloro-dl-phenylalanine and the colonies that grew were then counted (defined as thio^s^ apm^r^). To confirm curing of the delivery vectors, plates were replicated onto YMS medium containing 15 µg/ml of thiostrepton. After an incubation at 30 °C for 3 days, thiostrepton-resistant colonies were not observed on any plate. Transposition efficiency was calculated by the numbers of thio^s^ apm^r^/the numbers of thio^r^. The construction of pGM160∆*aac1::oriT::pheS*^*A339G/T278A*^::Tn*4556*-*aac(3)IV* containing perfectly matched IR-L was as follows: pUC19::Tn*4556*-*aac(3)IV* (derived from the *Bam*HI fragment from pUC1232; the largest fragment of *Nco*I/*Xho*I-pUC19::Tn*4556* was joined with 0.9-kb *Nco*I/*Sal*I-*aac(3)IV* prepared by PCR amplification) was digested with *Eco*RI/*Age*I and the largest 8.6-kb fragment was ligated with a 1.38-kb fragment containing perfectly matched IR-L and the N terminus region of ORF1 that was prepared by PCR amplification with pUC19::Tn*4556*-*aac(3)IV* as template DNA using the primer pair of forward: 5′-GTgaattcGAGCTCGGTACCCGGGGATCCGGATCGGG-3′ (lowercase characters indicate *Eco*RI site) and reverse: 5′-ACGGTGCCGAaccggtTGATCAGCATGGCCCGCC-3′ (lowercase characters indicate the *Age*I site). The largest 7.3-kb *Bam*HI-pUC19::Tn*4556*-*aac(3)IV* perfectly matched IR-L fragment was joined with the largest 8.4-kb *Bam*HI-digested pGM160∆*aac1::oriT::pheS*^*A339G/T278A*^ fragment and pGM160∆*aac1::oriT:: pheS*^*A339G/T278A*^::Tn*4556*-*aac(3)IV* perfectly matched IR-L was then obtained. The sequence of Tn*4556*-*aac(3)IV* perfectly matched IR-L has been deposited (accession # LC417442)

^a^Transposition efficiency was calculated from three independent experiments

## Conclusion

We corrected the Tn*4556* sequence by resequencing, and the most important ORFs for the class-II transposon, TnpA and TnpR, were accurately annotated. The new delivery vector was useful for the isolation of progeny carrying transpositions because a suicide gene encoding PheS^A339G/T278A^ as a counter-selectable marker eliminated the delivery vector by the selection of 4-chlorophenylalanine resistance after transposition. The replacement of the perfectly matched 38-bp IR-L variant enhanced transposition. Tn*4556*-*aac(3)IV* containing perfectly matched IR-L may be useful for transposon mutagenesis in* Streptomyces* strains.
